# Plan-Do-Study-Act in transcatheter aortic valve replacement

**DOI:** 10.1007/s12471-021-01559-6

**Published:** 2021-03-16

**Authors:** P. C. Smits

**Affiliations:** Department of Cardiology, Maasstad Hospital, Rotterdam, The Netherlands

Plan-Do-Study-Act is a model for improvement that provides a framework for developing, testing and implementing changes leading to improvement. It is also known as the Plan-Do-Check-Act cycle or Deming circle, named after its inventor William Edwards Deming, a management consultant in the 1950s.

The article by Van Steenbergen et al. in this issue of the *Netherlands Heart Journal* is a perfect example of how to set up (Plan) and implement an improvement process in the Cardiology Department (Do), how to evaluate this process (Study) and how to decide whether this improvement process will to be implemented (Act) [1]. The authors designed an outcome-based quality improvement strategy for patients who had been referred for transcatheter aortic valve replacement (TAVR) to the Catharina Hospital in Eindhoven, the Netherlands, by making changes to the selection process, pre-procedural workup, TAVR procedure and aftercare process.

From November 2015 onwards, all referred patients were seen pre-procedurally at a dedicated outpatient clinic, after which all of them underwent a dedicated computed tomography (CT) scan analysis and geriatric evaluation. Furthermore, from that moment on, all complex TAVR procedures were carried out by two dedicated operators, preferably under local anaesthesia, and the results were evaluated monthly. This study cohort of 532 patients was compared with a historical pre-quality strategy cohort (inclusion from January 2013 till October 2015) from the same hospital and with cohorts of TAVR patients registered in the Netherlands Heart Registration (NHR) from all other Dutch TAVR centres during the same study periods. A daring outcomes measurement was selected: peri-procedural mortality and all-cause mortality at 30 days and 1 year.

Compared with their own historical cohort of patients, Van Steenbergen et al. report an impressive relative reduction in all-cause mortality (ranging from 49 to 70%) after implementation of the quality improvement strategy in their institute. One can argue that after implementing a more stringent selection and workup process, probably fewer high-risk patients were selected and treated in the study cohort than in the historical cohort. However, looking at the patient characteristics of both cohorts, this does not seem to be the case at first glance. In both cohorts, the mean logistic European System for Cardiac Operative Risk Evaluation I (EuroSCORE I) was identical (on average 18), and in the study cohort, even more patients were treated with a logistic EuroSCORE I > 10.

Unfortunately, no information is provided on the number of patients who were turned down for TAVR after Heart Team consultation, nor on the number of patients with a logistic EuroSCORE I < 6 (medium to low risk) in both cohorts. It is natural to assume that after implementing a more stringent selection process, more patients will be turned down. It is good to know how often this occurred and what the outcomes of these rejected patients were. Furthermore, while more patients with a logistic EuroSCORE I > 10 were treated in the study cohort, this cohort ended up with the same mean logistic EuroSCORE I as the historical cohort. This can only indicate that more non–high-risk patients were treated in the study cohort as well, which can also explain the significant reduction in mortality.

As previously mentioned, Van Steenbergen et al. also put their data in a nationwide perspective by comparing the historical and study cohorts from the Catharina Hospital with the outcome database from the NHR, which consists of all other TAVR sites during the same study periods. Apart from the fact that their historical crude procedural mortality rates were already significantly higher than those in the average historical Dutch cohort, the most interesting observation is that—parallel to the impressive reduction in mortality in their hospital—the average 30-day mortality of all other Dutch TAVR sites also dropped significantly over time. One can wonder whether this great quality initiative was adopted by other Dutch TAVR centres as well or if other factors played a role.

**Fig. 1:**
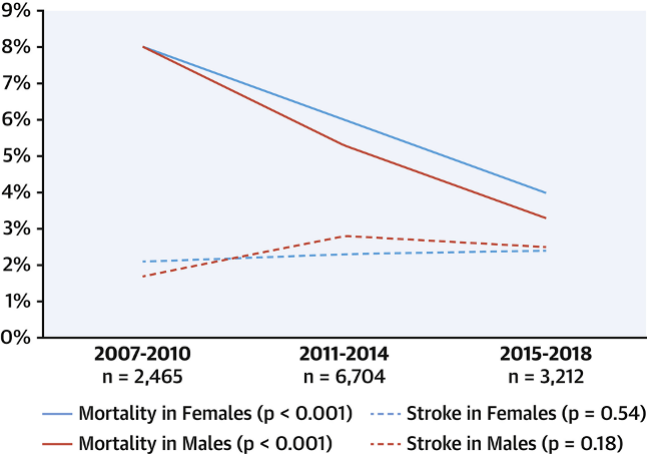
Temporal trends in 30-day outcomes after transcatheter aortic valve replacement procedures by sex from 2007–2018 (*N* = 12,381). (This figure was reprinted from [2]. Copyright, with permission from Elsevier)

A few things we know for sure are that in the past decade, when TAVR procedures came into use, many things have happened. Apart from increased skills, miniaturisation of the delivery system, usage of cerebral protection filters, development of retrievable valves, increased use of balloon expandable valves and better vascular closure techniques, more evidence became available for treating intermediate-risk patients with TAVR. The global temporal trends in 30-day outcomes of TAVR procedures from 2007 till 2018 have been nicely shown in an article by Vlastra et al. [2] In a large database with data of 12,381 TAVR patients included in 10 studies worldwide, 30-day mortality was reduced by 50% (from 8% to approximately 4%) in a decade of TAVR procedures (Fig. 1). In absolute and relative numbers, this is highly comparable to the 30-day mortality rates in the article by Van Steenbergen et al.

The question remains: What are the most important factors that optimise the outcome of TAVR procedures? It will probably be impossible to identify one or more discriminating factors, but it is more likely there is a chain of factors that have all contributed to the improved 30-day mortality rate. The most sobering fact, however, is that the incidence of stroke after TAVR remains constant, at around 2% (Fig. 1). Perhaps this should be the focus of the next outcome-based quality improvement strategy in TAVR patients.

Nevertheless, Van Steenbergen and colleagues are to be congratulated that by implementing a quality improvement strategy for TAVR procedures in their department, an impressive significant reduction in mortality was achieved. Another big compliment is the transparency this article is showing. In the world of today, we should not be afraid to openly share these important data with each other. We can only learn from one another and in the end, the patient benefits. The NHR is not only the way to measure, understand and compare outcomes of cardiac treatments between professionals in a safe and transparent surrounding, but also the way to improve.

The recent TAVR position and indication guideline documents from the Dutch Working Group of Transcatheter Heart Interventions describe a uniform indication process for TAVR procedures [3, 4]. By transforming the Heart Team decision process into parameters that are to be collected within the NHR, a complete and transparent dataset is created that can indicate which aortic valve disorder patients were or were not selected for TAVR and why. Only by measuring and reporting in a uniform and comprehensive manner, we can improve and make progress.
